# The Child Emotion Facial Expression Set: A Database for Emotion Recognition in Children

**DOI:** 10.3389/fpsyg.2021.666245

**Published:** 2021-04-29

**Authors:** Juliana Gioia Negrão, Ana Alexandra Caldas Osorio, Rinaldo Focaccia Siciliano, Vivian Renne Gerber Lederman, Elisa Harumi Kozasa, Maria Eloisa Famá D'Antino, Anderson Tamborim, Vitor Santos, David Leonardo Barsand de Leucas, Paulo Sergio Camargo, Daniel C. Mograbi, Tatiana Pontrelli Mecca, José Salomão Schwartzman

**Affiliations:** ^1^Developmental Disorders Program, Universidade Presbiteriana Mackenzie, São Paulo, Brazil; ^2^Hospital das Clínicas Faculty of Medicine, Universidade de São Paulo (USP), São Paulo, Brazil; ^3^Hospital Israelita Albert Einstein, São Paulo, Brazil; ^4^Fundación Universitaria Behaviour and Law, Madrid, Spain; ^5^Centro Universitário Internacional Uninter, São José do Rio Preto, Brazil; ^6^Verbal and Non-verbal Language Laboratory, Universidade Santa Úrsula, Rio de Janeiro, Brazil; ^7^Academia Militar das Agulhas Negras (AMAN), Resende, Brazil; ^8^Department of Psychology, Pontifícia Universidade Católica do Rio de Janeiro (PUC-RIO), Rio de Janeiro, Brazil; ^9^Department of Mental Health, Faculty of Medical Sciences, Santa Casa de São Paulo, São Paulo, Brazil

**Keywords:** emotion, emotional recognition, facial expression, child development, face processing

## Abstract

**Background:** This study developed a photo and video database of 4-to-6-year-olds expressing the seven induced and posed universal emotions and a neutral expression. Children participated in photo and video sessions designed to elicit the emotions, and the resulting images were further assessed by independent judges in two rounds.

**Methods:** In the first round, two independent judges (1 and 2), experts in the Facial Action Coding System, firstly analysed 3,668 emotions facial expressions stimuli from 132 children. Both judges reached 100% agreement regarding 1,985 stimuli (124 children), which were then selected for a second round of analysis between judges 3 and 4.

**Results:** The result was 1,985 stimuli (51% of the photographs) were produced from 124 participants (55% girls). A Kappa index of 0.70 and an accuracy of 73% between experts were observed. Lower accuracy was found for emotional expression by 4-year-olds than 6-year-olds. Happiness, disgust and contempt had the highest agreement. After a sub-analysis evaluation of all four judges, 100% agreement was reached for 1,381 stimuli which compound the ChildEFES database with 124 participants (59% girls) and 51% induced photographs. The number of stimuli of each emotion were: 87 for neutrality, 363 for happiness, 170 for disgust, 104 for surprise, 152 for fear, 144 for sadness, 157 for anger 157, and 183 for contempt.

**Conclusions:** The findings show that this photo and video database can facilitate research on the mechanisms involved in early childhood recognition of facial emotions in children, contributing to the understanding of facial emotion recognition deficits which characterise several neurodevelopmental and psychiatric disorders.

## Introduction

The ability to recognise and name one's own emotions and those of others according to facial expression clues is an important adaptive ability for both surviving and thriving in society. This ability is directly linked with the way an individual interacts with others and understands feelings and emotions in each context. This skill is even more important in childhood, when the first social interactions occur, before speech is fully developed (Izard, 2001).

A great deal of information can be determined at first glance in another person's face, such as age group, gender, and the direction of the gaze. Most non-verbal communication between humans is displayed on the face. Performed automatically and subjectively, facial analysis quickly informs a person about the emotions and behaviour of others during social interaction (Kanwisher and Moscovitch, 2000; Batty and Taylor, 2006).

Accurately decoding emotions from faces appears to be one of the main mechanisms for understanding social information. In ontogenetic research, important advances in facial emotion processing have been reported in the first year of life–for instance, new-borns look longer at smiling than neutral or fearful faces (Farroni et al., 2007; Rigato et al., 2011) and infants between 5 and 7 months show an attentional bias towards fearful faces (Leppänen and Nelson, 2012; Bayet and Nelson, 2019).

The advantages of understanding emotions for a child's healthy development are clear (Denham, 1998). Failure to recognise facial emotions is closely related to problems in child development. This failure is also characteristic of some developmental disorders (Happé and Frith, 2014) and may lead to delays in the primordial social skills necessary for adjusting to life in society. Poor emotion knowledge in children has been related to negative outcomes, including poor social functioning, poor academic performance, and internalising/externalising behaviour problems (Izard et al., 2001; Trentacosta and Fine, 2010; Ensor et al., 2011).

The scientific literature indicates that emotion recognition between 6 and 11 years of age predicts well-being and social relationships. Impaired emotional processing is related to increased vulnerability to developing mental disorders (Martins-Junior et al., 2011; Frith and Frith, 2012; Romani-Sponchiado et al., 2015). However, many of these disorders can occur before this point. Pre-school children aged 3 to 5 may suffer from an inability to integrate with their classmates and may avoid social activities, eat meals alone, not play with and not be accepted by their peers. All these difficulties could be related to problems with emotional identification (Herndon et al., 2013).

The onset of these social isolation symptoms and interactional difficulties indicates that psychological assessment is needed to diagnose disorders such as autism spectrum disorder, intellectual disability, conduct disorder, social anxiety, as well as to further characterise the emotion processing difficulties in specific genetic syndromes such as in autism spectrum (Frith and Frith, 2012). Furthermore, with the emergence and widespread application of new technologies such as eye tracking (Papagiannopoulou et al., 2014), there has been a sharp increase in basic and clinical research on the affective and cognitive neuroscience of face processing and emotion perception. For this reason, facial expression databases have been widely used in psychology, especially in studies of facial recognition and emotion recognition disorders (LoBue and Thrasher, 2015). However, only adult emotional facial stimuli are commonly used in these studies.

Recently, researchers have described the importance of having emotional expressions by children represented in databases to investigate the processing of these expressions during early development (Langner et al., 2010; Egger et al., 2011; LoBue and Thrasher, 2015). Therefore, there is a need for validated sets of child emotional faces for use in developmental research (Egger et al., 2011; Dalrymple et al., 2013).

When reviewing the emotion recognition databases in the literature (Haamer et al., 2017) it points out that an important choice in building a dataset is the way to arouse different emotions in the participants. That can be divided into three categories: posed, induced, and spontaneous expressions. A literature review was carried out for databases of images of children's facial expressions between 1999 and 2019, in PubMed/MEDLINE, using the following standardized controlled search terms: “facial stimuli set,” “children database,” “video database,” “facial emotional set,” “dynamic database,” “emotional facial expressions,” and “stimulus set.” Only six photographs databases of facial expressions of emotion could be found: Radboud Faces Database–RaFD (Langner et al., 2010), Child Emotions Picture Set–CEPS (Romani-Sponchiado et al., 2015), The Child Affective Facial Expression–CAFE (LoBue and Thrasher, 2015), National Institute of Mental Health Child Emotional Faces Picture Set–NINH-ChEFS (Egger et al., 2011), Child and Adolescent Dynamic Facial Expressions Stimuli Set–DuckEES (Giuliani et al., 2017), The Dartmouth Database of Children's Faces–DDCF (Dalrymple et al., 2013) (Table 1). Still, gaps remain in this area, as providing static as well as dynamic stimuli and having both posed and induced images. Indeed, only one of the existing databases offers video stimuli, DuckEES (Giuliani et al., 2017), resorting to the method of posed emotion, and without including preschool children. Regarding the video stimuli, dynamic stimuli provide greater naturalness and detail of the facial transformation process as an emotion is being expressed. Through videos, this process can be better understood and the moments when the facial expression reaches its peak can be selected with greater certainty (Krumhuber et al., 2017). Only one of the databases, the CAFE database (LoBue and Thrasher, 2015), depicts pre-school age children, although limited to posed stimuli. The pose method of inducing facial expression leads the person to carry out the emotion, providing an image to be replicated, or instructions to be followed indicating the person exactly the expression that is desired by the researcher. This method proves to be the easiest way to collect photographs of emotions, according to a review on emotion recognition performed by Haamer et al. (2017). The limitations found are the less authenticity of the stimuli and the lack of ecological validity. Often, because these expressions are not natural, they are exaggerated (Haamer et al., 2017). On the other hand, the method of induced photographs is able to capture more genuine emotions. The individual normally interacts with other people or watches audio-visual stimuli in order to evoke real emotions, thus generating more ecological stimuli and with less emphasis compared to the posed method. In the literature there is a lack of induced facial emotions databases with pre-school children. Given the importance of early diagnosis of developmental disorders and thorough characterization of associated social-emotional difficulties, more databases covering the pre-school age range using this induced photos and video stimuli should be produced. Thus, the present study aimed to develop an induced and posed, photo and video database of universal and neutral emotional expressions in Brazilian children between 4 and 6 years old.

**Table 1:** Published photo and video databases of children's facial expressions.

**Name**	**Age**	***N***	**Ethnicity**	***N* emotions**	**Type**	**Elicitation Method**	**Elicitation Technique**	***N* stimuli**	***N* of evaluators (validation)**	**Agreement**
RaFD	Unspecified	10	Caucasian	8	Photographs	Posed	Instructions	5,880	276 students	82%
NIMH-ChEFS	10–17	59	Caucasian or non-caucasian	6	Photographs	Posed	Instructions	482	20 volunteers	90.4%
DDCF	5–16	123	Caucasian	8	Photographs	Posed	Imagine a situation	Unspecified	163 students	70 ~ 90.6%
CAFE	2–8	154	17% Black, 30% Caucasian, 27% Asian, 17% Latino, and 9% other	7	Photographs	Posed	Children were asked to imitate expressions	1,192	100 students	66 to 81%
CEPS	6–11	18	14 Caucasians, 3 Blacks, and 1 Indigenous	7	Photographs	Posed and induced	Induced: videos, except for anger and sadness. Posed: mimic a photo expression	237	30 specialists	85%
DuckEES	8–18	37	Not identified	8	Videos	Unspecified	Imagine a situation	142	36 students	78 and 93%

*CAFE (LoBue and Thrasher, 2015), NINH Child Emotional Faces Picture Set–NINH-ChEFS (Egger et al., 2011), Child and Adolescent Dynamic Facial Expressions Stimuli Set–DuckEES (Giuliani et al., 2017) and Dartmouth Database of Children's Face–DDCF (Dalrymple et al., 2013)*.

## Materials and Methods

Children from 4 to 6 years of age were selected by convenience from a child acting agency in the city of São Paulo, Brazil. To ensure reliability, the parents or guardians were asked to declare their ethnicity (Caucasian, African, or Asian descent). A geneticist was consulted to perform an analysis of the children's photographs without knowledge of their names or any previous ethnicity statement. According to the parent's response and the geneticist's assessment, all participants were classified as being of Caucasian, African, or Asian descent.

This project was approved by the Research Ethics Committee of the Hospital Menino Jesus (number 048695/2017) and all research and methods were performed in accordance with relevant guidelines and regulations. The parents or guardians of all selected children provided written informed consent to participate and informed consent for disclosure of identifying images.

### Constructing the Child Emotion Facial Expression Set Database

Eliciting and capturing emotions in early childhood can be a challenging task, as stimuli must be carefully chosen. Five professionals from the Autism Spectrum Disorders Laboratory (Universidade Presbiteriana Mackenzie), in São Paulo, Brazil, were consulted to select cartoon excerpts targeted to elicit de induced emotions: happiness, anger, fear, disgust, surprise, sadness, contempt, or a neutral state. Regarding video capture, attention-getting age-appropriate cartoon excerpts were presented to the children. For inclusion of the video, at least two of the specialists needed to agree on the excerpt and choice of stimuli.

A pilot study was conducted to determine how to best elicit the target facial expressions, including media type (photos vs. videos), exposure duration, and sound stimuli. After a first pilot study (*n* = 4), some adjustments were made: the happiness, surprise, anger, disgust videos were changed because they did not evoke the emotion corresponding to what was expected. All videos were edited and some sound effects were added to certain sections so that the emotions elicited were enhanced. The final order of presentation of the videos followed a logic that did not incite a sequence of ambivalent emotions. In addition, it was determined that the order of the videos would begin with a neutral stimulus to create an atmosphere that would facilitate a child's adaptation period. After that, a second pilot study was conducted (*n* = 12), all videos were shortened to 1 min and 10 s. This approach was taken due to the young age of the children and the consequent difficulty in keeping them concentrated for 16 uninterrupted minutes.

To perform the posed stimuli, two methods were blended: facial expression and guided imagination. The children were invited to observe photographs and to perform the same facial expression as the child in the photograph. In addition, they received an activating phrase for each of them, for example: “You have just got a gift” (surprise), “You have just seen a ghost” (fear), and “You have lost your favourite toy” (sadness). These activating phrases were elaborated according to the children's age group. The phrases intend to make children revive or imagine a targeted situation from which the facial expression will occur. The video sequence was designed not to produce contradictory or ambivalent emotions, what could make the process of emotional expression difficult.

After the pilot studies, all the selected children came to the film studio (Universidade Presbiteriana Mackenzie) accompanied by their guardians. The children wore a white top and no makeup. The participants watched the cartoon excerpts in an unbroken sequence aimed at eliciting, respectively: neutrality, happiness, disgust, surprise, fear, sadness, anger, and contempt, according to following the universal emotions theory of Paul Ekman (Ekman and Friesen, 1971; Ekman and Heider, 1988). During this process the children were filmed, and these videos served as instruments for the analysis of experts (detailed below) for the production of photos and videos of spontaneous emotions. For posed photographs and videos, images of the previously mentioned emotions were obtained from the RaFD database (Langner et al., 2010) and were projected in the same sequence. Those images were used to facilitate children in carrying out emotional facial expression. The children were also filmed, generating video material for later analysis by experts (described below) and a construction of the images and videos posed emotions. A Panasonic HPX 370 camera was used for filming, and a 3200 Soft Light was used for the lighting system.

### Expert Analysis

Four judges certified in Ekman and Friesen's Facial Action Coding System (FACS) were involved in a multistep stimuli analysis and selection. The FACS is a method of analysis and score of emotional expression, quantifying important qualitative data. The certification is only given through an online test by Paul Ekman's Group. Firstly, judge 1 assessed the videos identifying the frames that most reliably represented each of the seven emotions and neutrality. A photo editing professional produced photographs and videos from the best frames of the videos selected by Judge 1. The videos and photographs were tagged and stored on the web. Only judge 2, 3 and 4 were blinded to the videos/ pictures used to elicit an emotional response in children, judge 1 had full access to the children faces and the sound of what they were hearing.

A second expert (judge 2), certified in Ekman and Friesen's Facial Action Coding System as well-analysed previously fragmented stimuli. Only images with 100% agreement in naming the facial emotions expression between the first (judge 1) and the second expert (judge 2) were included. It was the first round of analysis.

### ChildEFES Database Evidence of Validity

In order to compare the evaluation of the judges according to the features of the research subjects or the nature of the image, a stage of evidence of validity was performed through the analysis of two different evaluators (judges 3 and 4), who were also specialists in the Facial Action Coding System. It is important to consider that neither judge had participated in the previous steps and statistical analysis included the Kappa agreement index between the assessments of judges 3 and 4. According to Landis and Koch (1977), the most accepted arbitrary division for interpreting results is: Kappa <0.200 negligible; 0.210 to 0.400 minimum; 0.410 to 0.600 normal; 0.610 to 0.800 good; >0.810 excellent. The judges' accuracy in identifying the intended emotions was compared using the two-proportion equality test.

Therefore, a sub-analysis **ChildEFES** database was built using only images with 100% agreement among all four judges.

## Results

### Participant Selection Process

Among 182 children selected to participate in the study, 31 (17%) were excluded due to disagreement between the parents and the geneticist regarding the child's ethnic origin. Another three (2%) refused to participate in filming. Among the remaining 148 children, 16 were selected for the pilot study. Thus, 132 children (58% girls) participated in the database.

### Constructing the Database

With the number of 132 participants, a total of 29 h of video were captured in the studio. After assessment by judge 1, 3,668 stimuli were generated and classified. After judge 2's analysis, there was 100% agreement between Judges 1 and 2 regarding 1,985 stimuli (124 children, 55% girls), which were then selected for a second-round analysis with judges 3 and 4, in the phase of evidence of validity. In the agreement analysis of judges 3 and 4, an overall Kappa index of 0.70 (*p* < 0.001) and an agreement of 73% (1,447/1,985) were obtained for all database stimuli. This database was composed of 51% photographs (resolution 720 p), 49% videos (resolution 720 p) and 54% of all stimuli were induced. About ethnicity, 1,409 (71%) children were of Caucasian descent, 476 (24%) were of African descent and 99 (5%) were of Asian descent. Regarding distribution by age group, 744 (37%) stimuli were around 4-year-old, 609 (31%) around 5-year-old, and 632 (32%) around 6-year-old. The number of stimuli of each emotion were: neutrality 150, happiness 437, disgust 310, surprise 126, fear 269, sadness 183, anger 234, and contempt 276 (Table 6).

A comparison of the agreement between judges according to method of inducing (posed 78% vs. induced 70%, *p* < 0.01), type of stimulus (photography 74% vs. video 72%, *p* = 0.490), gender (female 73% vs. male 73%, *p* = 0.822), age (4 years 71%, 5 years 73% vs. 6 years 76%, *p* = 0.046 and *p* = 0.219, respectively), group and ethnicity (white 71%, black 76%, *p* = 0.026) is presented in Tables 2, 3. Significantly greater accuracy was found in posed stimuli than induced stimuli, and there was lower accuracy for children aged 4 years than those aged 6 years. No significant difference in agreement was found regarding gender or type of stimulus. Furthermore, there was greater accuracy in identifying the emotions of children of African descent than of children of Caucasian descent. In this analysis, children of Asian origin were excluded due to the small sample size.

**Table 2:** Agreement between judges 3 and 4 regarding facial expressions of different emotions according to image type, stimulus, gender, age and ethnicity.

**Categories**	**Kappa**	***p*-value (k)**	**Accuracy**			
			***N***	**Total**	**Accuracy %**	***p*-value**
**Image type**						
Photo	0.70	<0.001	746	1,014	73.6	0.490
Video	0.68	<0.001	701	971	72.2	
**Stimulus**						
Induced	0.63	<0.001	745	1,081	68.9	<0.001
Posed	0.74	<0.001	702	904	77.7	
**Gender**						
Female	0.69	<0.001	858	1,180	72.7	0.822
Male	0.69	<0.001	589	805	73.2	
**Age range**						
4 years	0.66	<0.001	527	744	70.8	0.046
5 years	0.68	<0.001	442	609	72.6	0.219
6 years	0.72	<0.001	478	632	75.6	Reference
**Ethnicity**						
White	0.67	<0.001	931	1,303	71.5	0.026
Black descent	0.76	<0.001	452	592	76.4	

**Table 3:** Agreement between judges 3 and 4 regarding facial expressions of different emotions according to induction method (posed or induced).

**Type**	**Kappa**	***p*-value (k)**	**Accuracy**			
			***N***	**Total**	**Accuracy %**	***p*-value**
**Photograph**						
Posed	0.73	<0.001	344	449	76.6	0.050
Induced	0.65	<0.001	402	565	71.2	
**Video**						
Posed	0.75	<0.001	358	455	78.7	<0.001
Induced	0.60	<0.001	343	516	66.5	

The percentage comparison of the judge's evaluation of the seven emotions plus neutrality is presented in Table 4. Happiness, disgust, and contempt had the highest agreement while neutrality and surprise had the lowest rates of agreement. In Table 5 is presented the total amount of stimuli evaluated by judges 3 and 4.

**Table 4:** Validation of ChildEFES database.

		**Judge 3**							
		**Contempt %**	**Happiness %**	**Fear %**	**Neutrality %**	**Disgust %**	**Anger %**	**Surprise %**	**Sadness %**
Judge 4	Contempt	9.27	0.76	0.05	0.60	0.25	0.55	0.00	0.91
	Happiness	0.50	17.78	0.71	0.71	0.65	0.20	0.20	0.55
	Fear	0.15	0.55	7.91	0.91	0.30	0.15	0.45	0.81
	Neutrality	1.11	0.35	0.25	6.50	0.20	0.50	0.55	2.47
	Disgust	0.20	0.91	0.10	0.30	9.77	0.40	0.05	0.71
	Anger	0.76	0.50	0.05	0.91	1.11	8.36	0.10	1.16
	Surprise	0.05	0.05	1.01	1.16	0.10	0.05	5.59	0.20
	Sadness	0.10	0.30	0.20	0.35	0.45	0.30	0.10	7.71

*Percentage comparison of judges' assessment of each emotion*.

**Table 5:** Number of stimuli evaluated for judge 3 and 4 of ChildEFES total database.

	**Contempt**	**Happiness**	**Fear**	**Neutrality**	**Disgust**	**Anger**	**Surprise**	**Sadness**	**Total**
Contempt	184	15	1	12	5	11	0	18	246
Happiness	10	353	14	14	13	4	4	11	423
Fear	3	11	157	18	6	3	9	16	223
Neutrality	22	7	5	129	4	10	11	49	237
Disgust	4	18	2	6	194	8	1	14	247
Anger	15	10	1	18	22	166	2	23	257
Surprise	1	1	20	23	2	1	111	4	163
Sadness	2	6	4	7	9	6	2	153	189
Total	241	421	204	227	255	209	140	288	1,985
**ChildEFE total**									
***n*** **= 1,985**									
	**Total of agreement judge 3 and 4**				**Total of stimuli ChildEFE total**			**% of Agreement**	
Contempt	184				276			67	
Hapiness	353				437			81	
Fear	157				269			58	
Neutrality	129				150			86	
Disgust	194				310			63	
Anger	166				234			71	
Surprise	111				126			88	
Sadness	153				183			84	

### Sub-analysis ChildEFES Database

**Figure 1:**
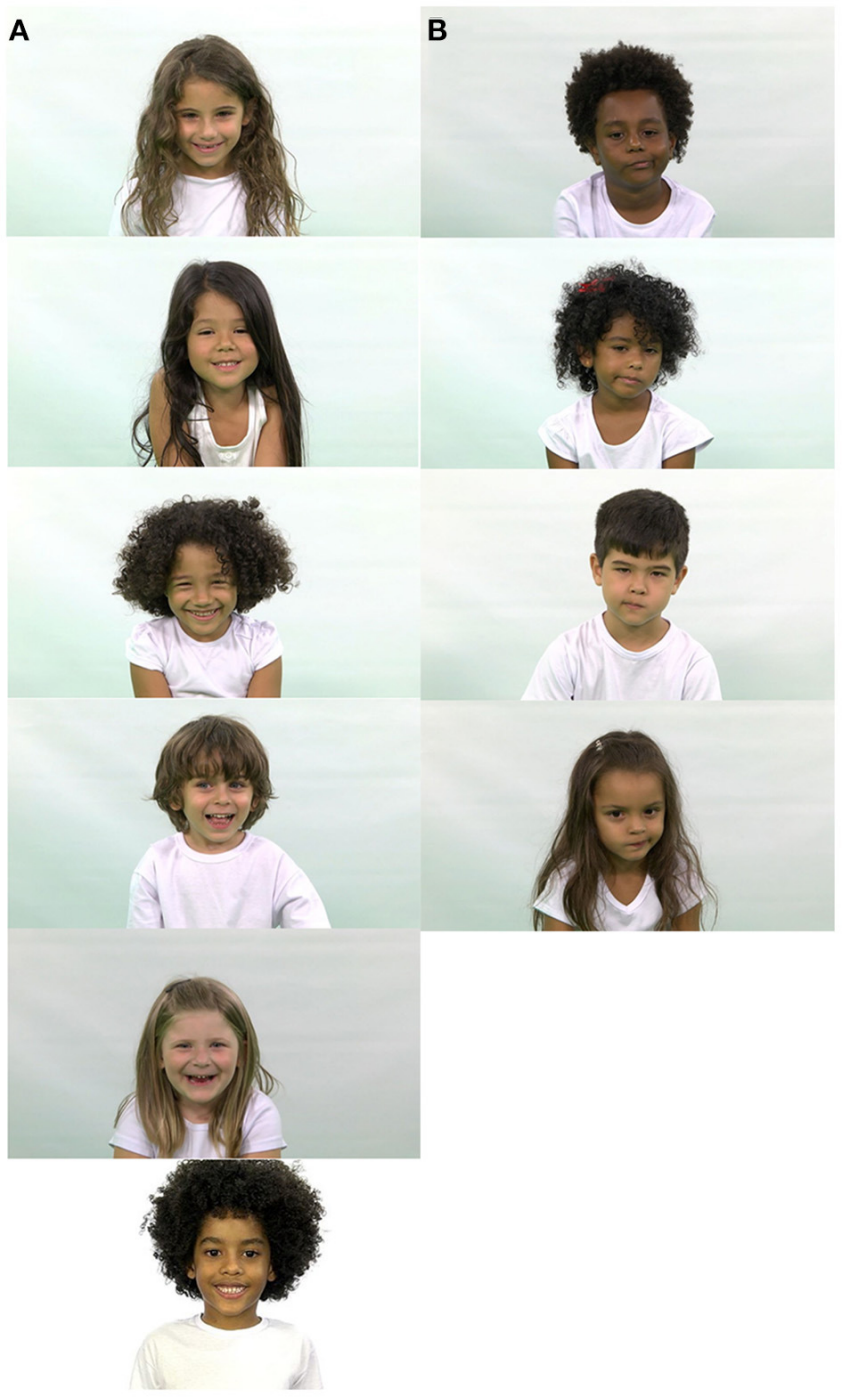
Examples of ChildEFES database images. **(A)** Happiness **(B)** Contempt.

**Figure 2:**
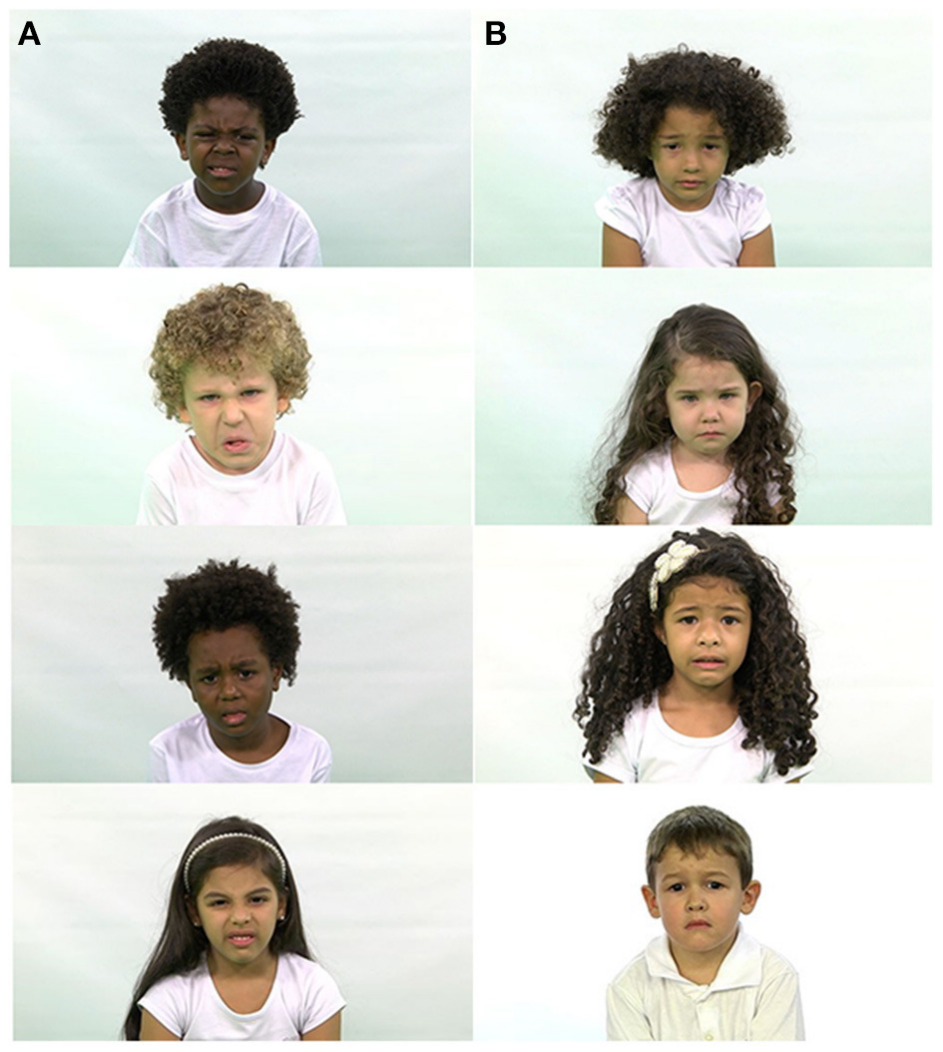
Examples of ChildEFES database images. **(A)** Disgust **(B)** Sadness.

**Figure 3:**
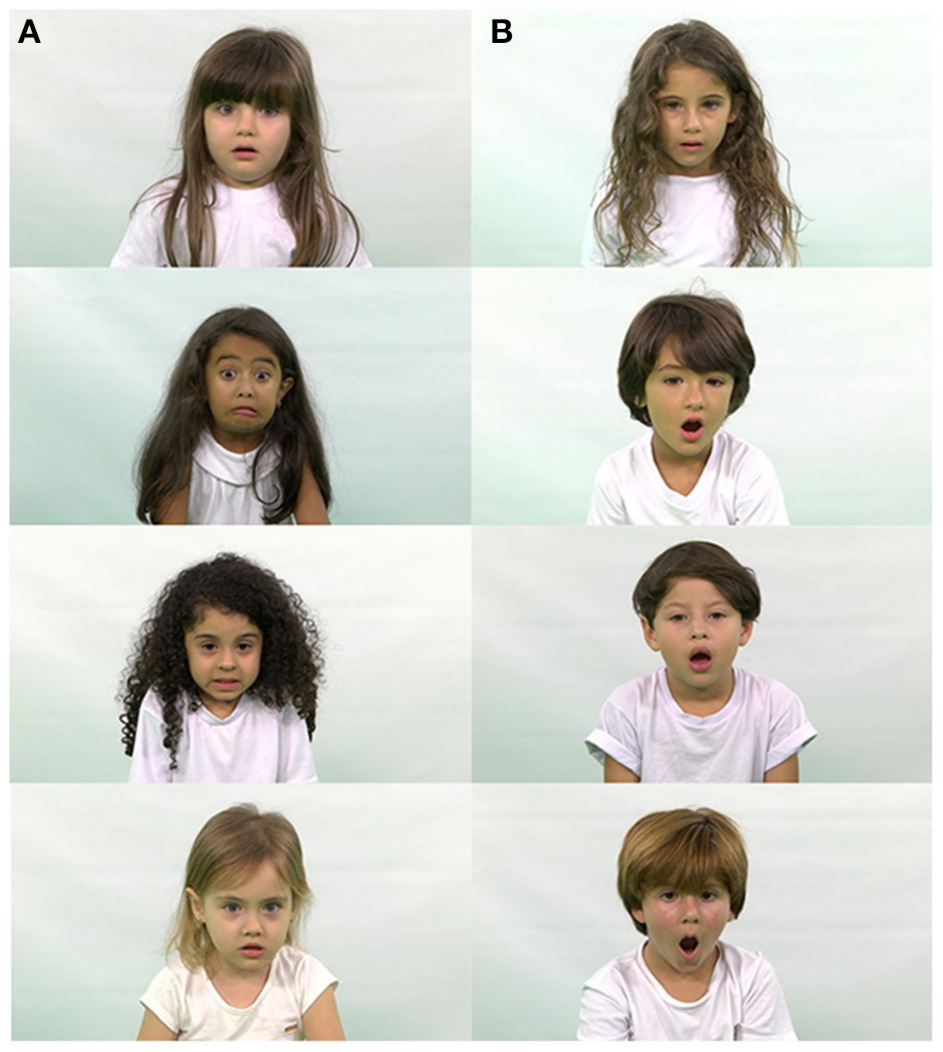
Examples of ChildEFES database images. **(A)** Fear **(B)** Surprise.

**Figure 4:**
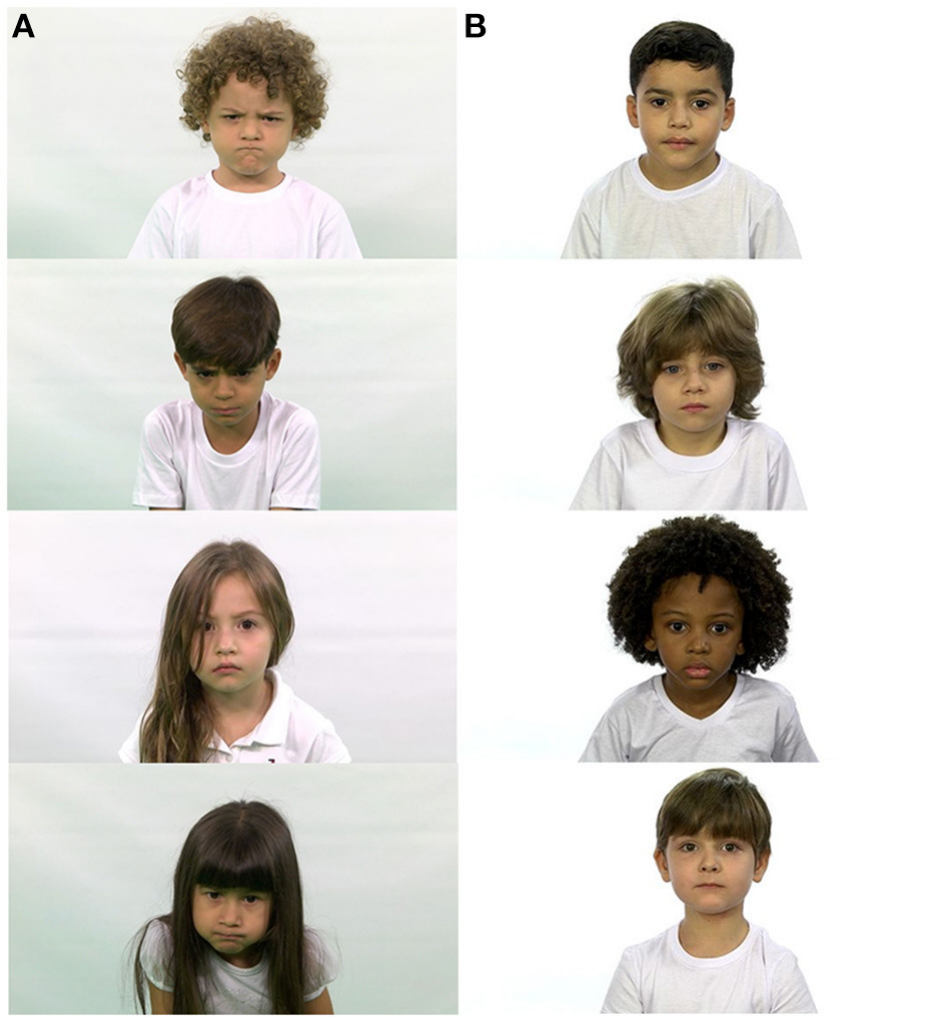
Examples of ChildEFES database images. **(A)** Anger **(B)** Neutral.

A sub-analysis with 100% agreement among four judges resulted in 1,381 stimuli from 124 participants (51% photographs, 51% induced), which compound the ChildEFES database (Figures 1–4). Among the selected stimuli, 563 (41%) came from boys, 818 (59%) from girls, 1,303 (66%) from children of Caucasian descent, 592 (30%) from children of African descent and 90 (4%) from children of Asian descent. Regarding format, 704 (51%) were photographs and 677 (49%) were videos. Concerning the method of expression induction, 679 (49%) stimuli were posed and 702 (51%) induced. Regarding distribution by age group, 405 (29%) stimuli were from 4-year-olds, 492 (36%) from 5-year-olds and 484 (35%) from 6-year-olds. The number of stimuli of each emotion were: neutrality 87 (59 induced, 42 videos), happiness 363 (298 induced, 170 videos), disgust 170 (87 induced, 99 videos), surprise 104 (22 induced, 52 videos), fear 153 (48 induced, 77 videos), sadness 144 (84 induced, 73 videos), anger 157 (38 induced, 68 videos), and contempt 183 (66 induced, 96 videos) (Table 6).

**Table 6:** Comparison of the distribution of photographs and videos by emotions from ChildEFE total vs. ChildEFE sub-analysis.

**Emotion**	**ChildEFE total**			**ChildEFE sub-analisis**		
	***n*** **= 1,985**			***n*** **= 1,381**		
	**Photographs**	**Videos**	**Total**	**Photographs**	**Videos**	**Total**
Contempt	130	146	276	87	96	183
Hapiness	223	214	437	193	170	363
Fear	142	127	269	76	77	153
Neutrality	72	78	150	45	42	87
Disgust	153	157	310	71	99	170
Anger	131	103	234	89	68	157
Surprise	66	60	126	52	52	104
Sadness	97	86	183	71	73	144
Total	1,014	971	1,985	684	677	1,361

## Discussion

Face databases are recognised as being of primary importance for emotional processing measurement in children. The published databases have some limitations, such as low representation of pre-school children, small number of induced stimuli and videos as main format.

Considering the published databases of child emotional expressions, it has been noticed that only one database—the CAFE (LoBue and Thrasher, 2015) predominantly studied facial expressions in the early childhood age group (up to 6 years of age). It is known that facial expressions of emotion can vary according to age, particularly in the first years of life. The DuckEES database contains dynamic stimuli in children from 8 to 18 years old (Giuliani et al., 2017), and has a greater representation of videos (142 posed videos) distributed relatively homogeneously by emotion. However, the DuckEES dataset did not include some of the universal emotions: (anger, contempt, surprise) and the all the stimuli were posed. In this order, the ChildEFE produced induced videos (971 total from posed and induced videos), which we believe is an important contribution.

Although the primary scope of this study was not to compare facial expression recognition among races, the judges had greater agreement when evaluating children of African descent than children of Caucasian descent. This suggests that both judges had greater ease in identifying facial expressions in this group and further research with different ethnicities should be important. In fact, ethnic differences in emotional recognition diminish with greater co-existence (Brigham et al., 1982; Carroo, 1986; Chiroro and Valentine, 1995), just as training can reduce the effects ethnicity on emotional recognition (Elliott et al., 1973; Goldstein and Chance, 1985). Studies with children and adolescents support the same hypothesis (Shepherd et al., 1981).

Regarding the method of stimulus inducement, there was greater agreement between the judges for posed stimuli than induced stimuli. This pattern remained when photo and video stimuli were analysed separately. This difference might be explained by the fact that posed stimuli generate exaggerated emotions, which ease identification. Moreover, videos and photos of induced emotions can involve more complex facial expressions, revealing subtle characteristics of the particular facial mimicry in each emotion.

In the literature, the only study involving children that also involved the two induction methods was the CEPS database (Romani-Sponchiado et al., 2015), which analysed 135 posed and 90 induced photographs. Unlike the present study, the CEPS database found no agreement differences between posed and induced photographs. However, this database had a much smaller number of stimuli and participants, which may have made this assessment less powerful.

It is important to point out that the greater the number of emotions evaluated, the more complex agreement becomes among judges. It should also be noted that the greatest agreement among the judges was for happiness. This result has also been observed in other child databases, such as RaFD (Langner et al., 2010), CEPS (Romani-Sponchiado et al., 2015), CAFE (LoBue and Thrasher, 2015), NINH-ChEFS (Egger et al., 2011), DuckEES (Giuliani et al., 2017), and DDCF (Dalrymple et al., 2013). Among the basic emotions, happiness is the only one with a positive valence, and recognition tends to be easier than for emotions with a negative valence. Relevant in social interactions, it is understood as an instrument of affective and social approximation.

As mentioned before, after an analysis of 100% agreement among four judges specialised in the Facial Action Coding System, the ChildEFES database obtained a relatively homogeneous distribution of stimuli in photos and videos, and of the nature of the stimulus (posed/induced). The facial expression of the main universal emotions and neutrality can be represented in photos, videos, in an induced and posed way.

The total database that contains 1985 stimuli represent a broader set of facial expressions with subtleties of each emotion. These differences may be due to less intense facial expressions or frames of an emotion at the beginning or end, which can generate disagreement among specialists (Kappa 0.70). However, these characteristics provide to the database higher ecological validity. The sub-analysis ChildEFES Database (100% agreement among experts), is composed of frames containing the expression of the most intense emotion or closest to its apex. In this way, both banks have their importance depending on the type of intervention proposed.

Some limitations of this study should be considered: firstly, a secondary analysis performed by a larger group untrained in the Facial Action Coding System would be of interest. Since the database is designed for assessment of emotion recognition abilities in children, a second study should be considered to validate stimuli with children playing the role of judges. Secondly, in this study there was a predominance of Caucasians, the small number of participants from other ethnic groups did not allow a detailed comparison of ethnic heterogeneity influence on emotion recognition agreement. Thirdly, the small number of stimuli represented by children of Asian origin may be a limitation for the use of this database in this population. And finally, all the children were from southern Brazil, which limited the range of ethnicity and facial types.

The ChildEFES database includes a greater range of emotions, static and dynamic stimuli, ethnic variability and young age. This instrument, whose website is under construction, will be available online. We believe it will be an helpful instrument to facilitate future research on social-emotional processing and may assist diagnostic and intervention efforts for developmental disorders in clinical practice.

## Data Availability Statement

The original contributions presented in the study are included in the article/supplementary material, further inquiries can be directed to the corresponding author/s.

## Ethics Statement

This project was approved by the Research Ethics Committee of the Hospital Menino Jesus (number 048695/2017). Written informed consent to participate in this study was provided by the participants' legal guardian/next of kin. Written informed consent was obtained from the minor(s)' legal guardian/next of kin for the publication of any potentially identifiable images or data included in this article.

## Author Contributions

JN: conception and design, analysis and interpretation, data collection, writing the article, critical revision of the article, final approval of the article, statistical analysis, and overall responsibility. AO: conception and design, critical revision of the article, and final approval of the article. RS: conception and design, analysis and interpretation, critical revision of the article, final approval of the article, and statistical analysis. VL: data collection, and critical revision of the article. EK, MD'A, DM, and TM: critical revision of the article. AT, VS, DdL, and PC: data collection. JS: conception and design, CAPES-Proex (CAPES/Proex Grant No. 0653/2018), and MACKPESQUISA research funding. All authors have read and approved the final version of the article.

## Conflict of Interest

The authors declare that the research was conducted in the absence of any commercial or financial relationships that could be construed as a potential conflict of interest.

## References

[B1] Batty M. Taylor M. J. (2006). The development of emotional face processing during childhood. Dev. Sci. 9, 207–220. 10.1111/j.1467-7687.2006.00480.x 16472321

[B2] Bayet L. Nelson C. A. (2019). The perception of facial emotion in typical and atypical development, in Handbook of Emotional Development, eds LoBue V. Pérez-Edgar K. Buss K. V. (Cham: Springer Nature Switzerland). 10.1007/978-3-030-17332-6_6

[B3] Brigham J. C. Maass A. Snyder L. D. Spaulding K. (1982). Accuracy of eyewitness identification in a field setting. J. Pers. Soc. Psychol. 42, 673–681. 10.1037/0022-3514.42.4.673

[B4] Carroo A. W. (1986). Other race recognition: a comparison of black American and African subjects. Percept. Mot. Skills 62, 135–138. 10.2466/pms.1986.62.1.135 3960654

[B5] Chiroro P. Valentine T. (1995). An investigation of the contact hypothesis of the own-race bias in face recognition. Q. J. Exp. Psychol. A 48, 879–894.

[B6] Dalrymple K. A. Gomez J. Duchaine B. (2013). The dartmouth database of children's faces: acquisition and validation of a new face stimulus set. PLoS ONE. 8:e79131. 10.1371/journal.pone.0079131 24244434 PMC3828408

[B7] Denham S. A. (1998). Emotional Development in Young Children. New York, NY: Guilford.

[B8] Egger H. L. Pine D. S. Nelson E. Leibenluft E. Ernst M. Towbin K. E.. (2011). The NIMH child emotional faces picture set (NIMH-ChEFS): a new set of children's facial emotion stimuli. Int. J. Methods. Psychiatr. Res. 20, 145–156. 10.1002/mpr.343 22547297 PMC3342041

[B9] Ekman P. Friesen W. V. (1971). Constants across cultures in the face and emotion. J. Pers. Soc. Psychol. 17, 124–129. 10.1037/h0030377 5542557

[B10] Ekman P. Heider K. G. (1988). The universality of a contempt expression: a replication. Motiv. Emot. 12, 303–308. 10.1007/BF00993116

[B11] Elliott E. S. Wills E. J. Goldstein A. G. (1973). The effects of discrimination training on the recognition of white and oriental faces. Bull. Psychon. Soc. 2, 71–73. 10.3758/BF03327717

[B12] Ensor R. Spencer D. Hughes C. (2011). ‘You Feel Sad?’ Emotion understanding mediates effects of verbal ability and mother–child mutuality on prosocial behaviors: findings from 2 years to 4 years. Soc. Dev. 20, 93–110. 10.1111/j.1467-9507.2009.00572.x

[B13] Farroni T. Menon E. Rigato S. Johnson M. H. (2007). The perception of facial expressions in newborns. Eur. J. Dev. Psychol. 4, 2–13. 10.1080/17405620601046832 20228970 PMC2836746

[B14] Frith C. D. Frith U. (2012). Mechanisms of social cognition. Annu. Rev. Psychol. 63, 287–313. 10.1146/annurev-psych-120710-100449 21838544

[B15] Giuliani N. R. Flournoy J. C. Ivie E. J. Von Hippel A. Pfeifer J. H. (2017). Presentation and validation of the DuckEES child and adolescent dynamic facial expressions stimulus set. Int. J. Methods. Psychiatr. Res. 26:e1553. 10.1002/mpr.1553 28090698 PMC6877251

[B16] Goldstein A. G. Chance J. E. (1985). Effects of training on Japanese face recognition: reduction of the other-race effect. Bull. Psychon. Soc. 23, 211–214. 10.3758/BF03329829

[B17] Haamer R. E. Rusadze E. Lsi I. Ahmed T. Escalera S. Anbarjafari G. (2017). Review on emotion recognition databases. Hum. Robot. Interact. Theor. Appl. 3, 39–63. 10.5772/intechopen.72748

[B18] Happé F. Frith U. (2014). Annual research review: towards a developmental neuroscience of atypical social cognition. J. Child. Psychol. Psychiatry 55, 553–557. 10.1111/jcpp.12162 24963529

[B19] Herndon K. J. Bailey C. S. Shewark E. A. Denham S. A. Bassett H. H. (2013). Preschoolers' emotion expression and regulation: relations with school adjustment. J. Genet. Psychol. 174, 642–663. 10.1080/00221325.2012.759525 24303577 PMC3856321

[B20] Izard C. Fine S. Schultz D. Mostow A. Ackerman B. Youngstrom E. (2001). Emotion knowledge as a predictor of social behavior and academic competence in children at risk. Psychol. Sci. 12, 18–23. 10.1111/1467-9280.00304 11294223

[B21] Izard C. E. (2001). Emotional intelligence or adaptive emotions? APA PsycArticles 1, 249–257. 12934684

[B22] Kanwisher N. Moscovitch M. (2000). The cognitive neuroscience of face processing: an introduction. Cogn. Neuropsychol. 17, 1–11. 10.1080/026432900380454 20945168

[B23] Krumhuber E. G. Skora L. Küster D. Fou L. (2017). A review of dynamic datasets for facial expression research. Emot. Rev. 9, 280–292. 10.1177/1754073916670022

[B24] Landis J. R. Koch G. G. (1977). The measurement of observer agreement for categorical data. Biometrics 33, 159–174. 843571

[B25] Langner O. Dotsch R. Bijlstra G. Wigboldus D. H. Hawk S. T. Van Knippenberg A. D. (2010). Presentation and validation of the radboud faces database. Cogn. Emot. 24, 1377–1388. 10.1080/02699930903485076

[B26] Leppänen J. M. Nelson C. A. (2012). Early development of fear processing. Curr. Dir. Psychol. Sci. 21, 200–204. 10.1177/0963721411435841

[B27] LoBue V. Thrasher C. (2015). The child affective facial expression (CAFE) set: validity and reliability from untrained adults. Front. Psychol. 5:1532. 10.3389/fpsyg.2014.01532 25610415 PMC4285011

[B28] Martins-Junior F. E. Sanvicente-Vieira B. Grassi-Oliveira R. Brietzke E. (2011). Social cognition and theory of mind: controversies and promises for understanding major psychiatric disorders. Psychol. Neurosci. 4, 347–351. 10.3922/j.psns.2011.3.008

[B29] Papagiannopoulou E. A. Chitty K. M. Hermens D. F. Hickie I. B. Lagopoulos J. (2014). A systematic review and meta-analysis of eye-tracking studies in children with autism spectrum disorders. Soc. Neurosci. 9, 610–632. 10.1080/17470919.2014.934966 24988218

[B30] Rigato S. Menon E. Johnson M. H. Farroni T. (2011). The interaction between gaze direction and facial expressions in newborns. Eur. J. Dev. Psychol. 8, 624–636. 10.1080/17405629.2011.602239 PMC2836746 20228970

[B31] Romani-Sponchiado A. Sanvicente-Vieira B. Mottin C. Hertzog-Fonini D. Arteche A. (2015). Child emotions picture set (CEPS): development of a database of children's emotional expressions. Psychol. Neurosci. 8, 467–478. 10.1037/h0101430

[B32] Shepherd J. W. Davies G. Ellis H. D. (1981). Studies of cue saliency, in Perceiving and Remembering Faces, eds Davies G. Ellis H. D. Shepherd J. W. (New York, NY: Academic Press), 105–131.

[B33] Trentacosta C. J. Fine S. E. (2010). Emotion knowledge, social competence, and behavior problems in childhood and adolescence: a meta-analytic review. Soc. Dev. 19, 1–29. 10.1111/j.1467-9507.2009.00543.x 21072259 PMC2975582

